# Pyrosequencing reveals a shift in symbiotic bacteria populations across life stages of *Bactrocera dorsalis*

**DOI:** 10.1038/srep09470

**Published:** 2015-03-30

**Authors:** Awawing A. Andongma, Lun Wan, Yong-Cheng Dong, Ping li, Nicolas Desneux, Jennifer A. White, Chang-Ying Niu

**Affiliations:** 1 College of Plant Science & Technology, Huazhong Agricultural University, Wuhan 430070, China; 2 Pest Control Division, National Agricultural Technology Extension and Service Center, Ministry of Agriculture, Beijing 100125, China; 3 French National Institute for Agricultural Research (INRA), UMR1355-ISA, 06903 Sophia-Antipolis, France; 4 Department of Entomology, University of Kentucky, Lexington, KY 40546-0091, USA

## Abstract

*Bactrocera dorsalis* is one of the most economically important fruit flies around the world. In this study, 454 pyrosequencing was used to identify the bacteria associated with different developmental stages of *B. dorsalis*. At ≥ 97% nucleotide similarity, total reads could be assigned to 172 Operational Taxonomic Units belonging to six phyla. Proteobacteria dominated in immature stages while Firmicutes dominated in adult stages. The most abundant families were *Enterococcaceae* and *Comamondaceae*. The genus *Comamonas* was most abundant in pupae whereas completely absent in adults. Some identified species had low sequence similarity to reported species indicating the possibility of novel taxa. However, a majority sequence reads were similar to sequences previously identified to be associated with *Bactrocera correcta*, suggesting a characteristic microbial fauna for this insect genus. The type and abundance of different bacterial groups varied across the life stages of *B. dorsalis*. Selection pressure exerted by the host insect as a result of its habitat and diet choices could be the reason for the observed shift in the bacteria groups. These findings increase our understanding of the intricate symbiotic relationships between bacteria and *B. dorsalis* and provide clues to develop potential biocontrol techniques against this fruit fly.

The insect gut contains an array of micro-organisms that influences its health and fitness^1^,^2^. In the absence of symbiotic bacteria, insects have been reported to have a reduced growth rate and high mortality^2^,^3^. Insects acquire these bacteria by horizontal and/or vertical transmission patterns^4^,^5^,^6^. Some of these symbionts have been shown to carry out diverse roles in their relationship with insects which range from egg production, insect development, survival and fitness^1^,^7^,^8^,^9^. This may also be a similar case across the developmental stages of the oriental fruit fly. *Enterobacter cloacae*, *Bacillus cereus* and *Citrobacter feu*ndii for example has been shown to act as an attractive lure for the oriental fruitfly^10^. However there is still a huge gap of knowledge on the roles several symbiotic bacteria play in their relationship with *Bactrocera dorsalis*.

The symbiotic relationship between insects and bacteria is becoming more obvious and may provide a promising strategy for biological control of insect pests. Unfortunately, there has been a gap in the determination of the number and range of these bacteria symbionts associated with insects^1^,^11^. This has been linked to inaccuracy and inefficiency in the estimation technique which could lead to over estimation or under estimation of bacteria groups and abundance^1^. Culturing techniques, for example, do not take into consideration uncultivable bacteria and the pH required for optimal growth of each species. As a result, the pH of the growth media may determine the type and rate of bacterial growth leading to inaccuracy in the estimation of bacteria diversity. Other advanced techniques of bacterial identification based on the 16s rRNA gene used in the past have also greatly improved our understanding of insect gut bacteria. These include Polymerase Chain Reactions (PCR) targeting genes, molecular fingerprinting techniques, and oligonucleotide probe-based hybridization techniques. However, a host of limitations limitations are associated with these techniques, including the inability to provide detailed information concerning the microbial species present in the insect gut, the gene and pathways for different biological processes^12^. For example, the gene targeting PCR technique is dependent on existing sequence information to design primers for PCR amplification and clones only partial sequence of a gene^13^. DGGE on the other hand has been shown to give variable results that does not reflect the real picture^14^. In addition, technical problems and the gut environment may interfere with the detection of bacteria when the FISH method is used^15^. In order to understand how best bacteria symbionts could be used for insect control, it is important to understand in detail the bacterial diversity associated with each developmental stage. 454 GS FLX pyrosequencing technique enables such a study^12^ and gives us an opportunity to collect a more detailed sampling data^16^. Even though this technique, too, has drawbacks^16^,^17^, it provides a simple and cost-effective mechanism for characterizing bacterial community composition, particularly when analyzed conservatively to yield a reliable data set^18^.

*Bactrocera dorsalis* is a menace to horticultural industry around the world^19^,^20^. In recent years, the intricate relationship between fruit flies and their symbiotic bacteria has been explored as a strategy to control these pests. In order for this strategy to be efficiently exploited, it is important to develop a comprehensive understanding of the bacterial community structure. However, the bacterial symbionts associated with the different life stages of *B. dorsalis* still remained unclear. The aim of this work was therefore to determine the diversity of gut bacteria associated with the life stages of *B. dorsalis* using 454 GS-FLX pyrosequencing of PCR generated amplicons of the 16S rRNA gene. Wild populations of *B. dorsalis* were used for this experiment. Bacteria were sampled from all developmental stages of the fly in an attempt to understand the diversity of bacteria associated with this pest and how they vary across its life cycle.

## Results

### Diversity Estimation

The total number of reads obtained from the 6 samples was 66,527. After removal of chimeric sequences and mismatches, 46,332 sequences remained, which corresponded to 731 unique sequences. These sequences were clustered into 172 operational taxonomic units (OTU) with at most 3% dissimilarity in nucleotide identity. The taxonomy and abundance of all the 172 OTUs can be found as supplementary Table S1 online. Rarefaction curves for each sample do not asymptote, indicating that additional rare bacterial taxa are likely present in each sample. ACE and Chao1 diversity estimators calculated using a random set of sequences of equal sampling intensity across the life stages, suggest that substantial additional OTUs are likely to be present in some life stages, although coverage estimates were very high for all samples (Table 1). Both Shannon and Simpson diversity indices, which incorporate evenness as well as species richness, suggest that taxonomic diversity of the bacterial community is higher in egg and larval stages than pupal and adult stages of *B. dorsalis*.

**Figure 1:**
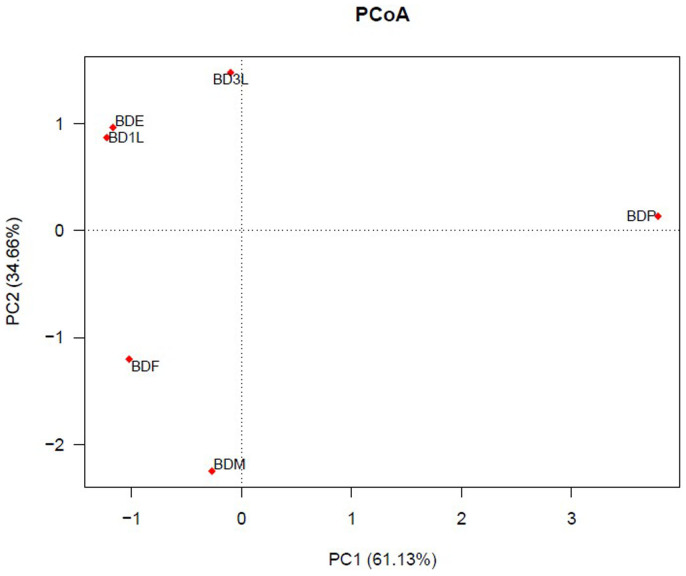
Comparism of bacteria community in samples from different developmental stages of *Bactrocera dorsalis*. Principal Coordinate Analysis (PCoA) was generated with OTUs (at 97% similarity) present in the different clone libraries; BDE- *Bactrocera dorsalis* egg, BD1L- *Bactrocera dorsalis* first instar larva, BD3L- *Bactrocera dorsalis* third instar larva, BDP- *Bactrocera dorsalis* pupa, BDF- *Bactrocera dorsalis* female, BDM- *Bactrocera dorsalis* male.

**Figure 2:**
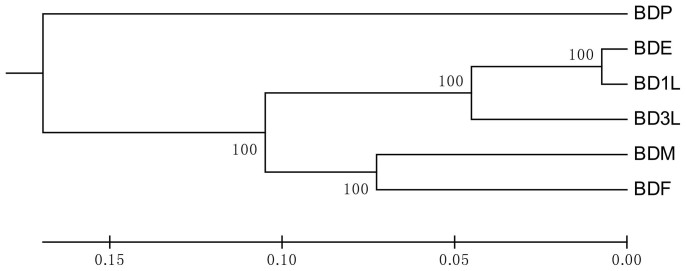
Dendrogram showing the relationship of bacteria groups associated with different life stages of *Bactrocera dorsalis*: Jacknifed support values of 100 are shown.

Our experimental results revealed that the oriental fruit fly hosts a wide diversity of symbiotic bacteria with variation across its life stages. The extent of variation of symbiotic bacteria population in the different life stages correlates with the natural habitat of each stage. The bacteria diversity in the *Bactrocera dorsalis* egg (BDE) was similar to the *Bactrocera dorsalis* first instar larva (BD1L) and these two are closely related to the *Bactrocera dorsalis* third instar larva (BD3L). Bacterial diversity in the *Bactrocera dorsalis* male (BDM) and *Bactrocera dorsalis* female (BDF) adult groups were closely related. The *Bactrocera dorsalis* pupa (BDP) was distantly related to the other developmental stage (Fig. 1 and 2).

### Taxonomic distribution of bacteria identified by pyrosequencing

**Figure 3:**
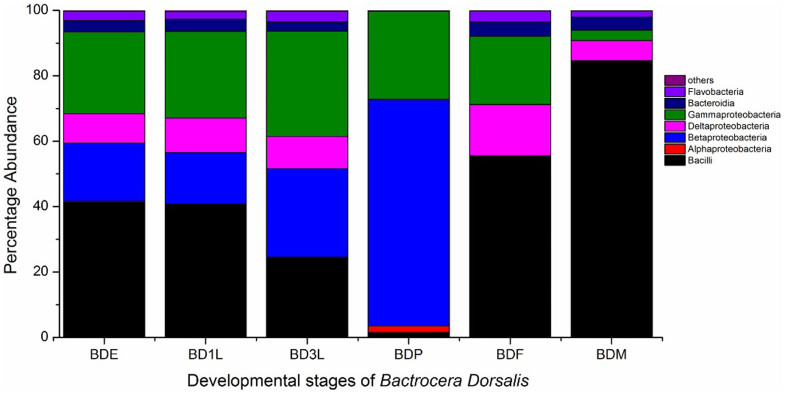
Relative bacteria composition of different bacteria classes in the guts of different developmental stage of *Bactrocers dorasalis*: BDE- *Bactrocera dorsalis* egg, BD1L- *Bactrocera dorsalis* first instar larva, BD3L- *Bactrocera dorsalis* third instar larva, BDP- *Bactrocera dorsalis* pupa, BDF- *Bactrocera dorsalis* female, BDM- *Bactrocera dorsalis* male.

Operational taxonomic units were further assigned to different taxa and their relative taxonomic abundance was estimated across the different life stages. A total of 12 known bacteria classes belonging to some 6 known phyla were identified in addition to some unknown groups; these were Firmicutes, Proteobacteria, Bacteriodetes, Actinobacteria, Fusobacteria and Deinococcus- Thermus. Of these, only the Firmicutes, Proteobacteria and Bacteriodetes were represented in all life stages and represented by a large number of reads. The Firmicutes and the Proteobacteria represented the highest number of reads in each life stage, together making up at least 92% of the total bacteria population in each life stage (Fig. 3). During the insect life cycle the bacteria population shifts from Proteobacteria, which dominated in the developmental stages, to Firmicutes, which dominated in the adult stages.

### Firmicutes

**Figure 4:**
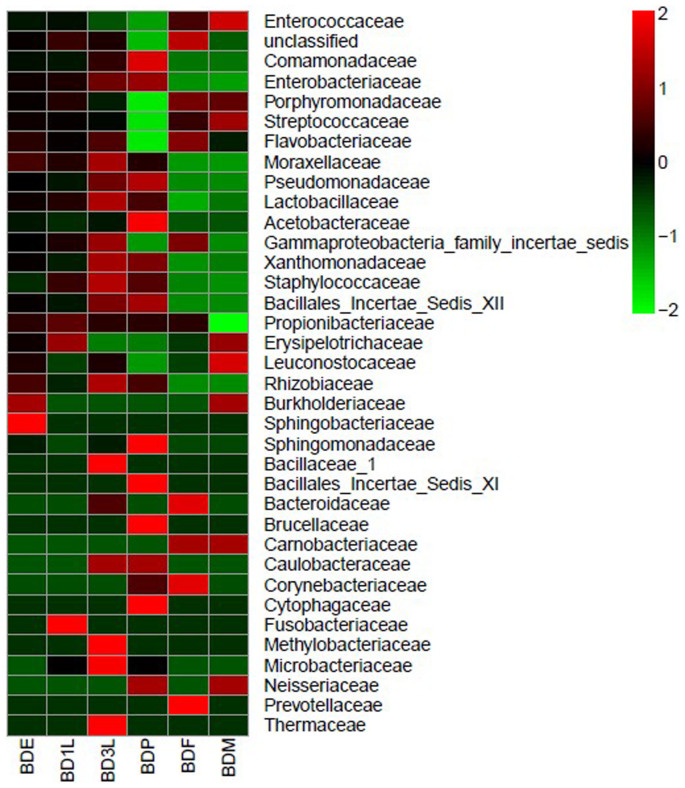
Heat maps showing bacterial family frequency distribution across the six different life stages. The different color intensities represent the relative bacteria abundance in each life stage. The clustering within each bacteria group (Y-axis) is based on sequence similarity of bacteria reads present in each life stage (x-axis).

Sequence reads belonging to the Firmicutes phylum could be classified into the classes Bacilli and Erysipelotrichia in addition to some unidentified classes. Across the different life stages, Erysipelotrichia was generally represented by very low reads and were completely absent in the third instar larva and pupae. The Bacilli class bore the most abundant reads in all life stages except the pupae and the third instar larva (Fig. 3). These reads mostly belong to the family *Enterococcaceae* (Fig. 4). The Bacilli class was dominated by 2 OTU that were related to the species *Enterococcus haemaperoxidus* and *Lactococcus latis*. A BLASTn search of the consensus sequence for the *Enterococcus* OTU in NCBI was most similar (≥97%) to a bacterium present in the gut of *Bactrocera correcta* (Genbank accession number JQ950506), while the *Lactococcus* OTU was most similar to uncultured environmental bacteria (Genbank accession number AB828413). The *Enterococcus* OTU had the most abundant reads in all life stages except the pupa, comprising 79% of the total OTUs present in the adult male, 51% of the adult female's population and barely represented by a few reads in the pupal stage (Table 2). On the other hand, reads related to the *Lactococcus* OTU represented at least 3% of the total bacteria symbionts in all life stages, and was absent from the pupae.

### Proteobacteria

Reads belonging to the Proteobacteria could be assigned to 4 classes in addition to some unidentified groups. These classes were the Alphaproteobacteria, Betaproteobacteria, Deltaproteobacteria and Gammaproteobacteria. Alphaproteobacteria was present only in the immature stages and completely absent in the adults. Betaproteobacteria had relatively high reads in the immature stages, particularly the pupae, but were almost absent in the adults (Fig. 3). Betaproteobacteria was represented by 3 families, namely *Comamonadaceae*, *Burkholderiaceae* and *Neisseriaceae* in addition to some unclassified groups. However, the majority of the reads belong to the *Comamonadaceae* (Fig. 4) which was mainly represented by the second most common OTU, with sequence reads similar to *Comamonas terrigena*. This OTU was most similar to uncultivated environmental bacteria (Genbank accession number JF692613, KF785066) (Table 2). This OTU was the most abundant bacterium in all life stages except third instar larvae and non-feeding pupae. In pupae, this bacterium was represented only by very few reads.

The Deltaproteobacteria represented about 10% of the total reads in almost all life stages except the pupa with only a very few reads. These reads were dominated by a single OTU in the order Desulfovibrionales that could not be assigned to any known family or genus. These sequence reads were similar to a bacterium associated with the gut of congeneric *B. correcta* (Genbank accession number JQ950461). The Gammaproteobacteria had at least 20% of the total reads in all life stages except the adult male, where it comprised only 3% of the reads (Fig. 3). This class included bacteria from the families *Enterobacteriaceae*, *Pseudomonadaceae* and *Moraxellaceae*, in addition to a Gammaproteobacterial OTU, which was present in all stages and comprised more than 12% of bacterial reads in all life stages except the adult male and pupae, but could not be classified even to order. This most common OTU was also most similar (99%) to a bacterium from the gut of *B. correcta* (Genbank accession number JQ950479). The other common Gammaproteobacteria OTUs were similar to *Enterobacter aerogenes* (FJ393309.1), *Psedomonas indica* (AF302795.1) and *Acinebacter junii* (FJ544392.1). However the best matches of the last two strains were most similar to a bacterium that has identified in *B. correcta* and frog respectively. Bacteria belonging to the Gammaproteobacterial group were either rare or absent from adult stages of *B. dorasalis* (Table 2).

### Bacteroidetes

Members of the Bacteroidetes phylum were also present in all life stages of *B. dorsalis*. These bacteria were primarily represented by the classes Bacteroidia and Flavobacteria. OTU in these classes generally were found across all life stages, but had less than 4% of the reads in each life stage (Table 2). Sequence reads belonging to this phylum frequently could not be assigned to any known genera. However, sequence reads corresponding to the most common OTU were once again found to be most similar to bacteria associated with the gut bacteria of *B. correcta* (Genbank accession number JQ950512, JQ950459) or to other insect gut associates, at a lower level of similarity (93–98%; Genbank accession numbers JN680576, JX457996, KC865714) (Table 2).

### Other Phyla

Other bacterial phyla associated with *B. dorsalis* were found to be present only in some life stages and when present, only having less than 0.2% of the total reads in each life stage. Actinobacteria was represented by 3 families, *Propionibacteriaceae*, *Microbacteriaceae* and *Corynebacteriaceae*. Fusobacteria was represented only by the family *Fusobacteriaceae*, present only in the first instar larva and had only 0.01% of the total reads. Similarly Deinococcus-Thermus was found only in the third instar larva where it composed 0.03% of the total reads and was represented only by a single family, Thermaceae. Lastly, there were groups of sequences that were distantly related to all known phyla. These OTUs were not found in all life stages, and they invariably had low prevalence.

## Discussion

This study presents a culture independent analysis of the bacteria associated with the gut of different life stages of *B. dorsalis*. To the best of our knowledge, this is the first documentation of the symbiotic bacteria present in the immature stages of *B. dorsalis*. In this study we found that *B. dorsalis* appears to have a relatively higher diversity of bacterial symbionts when compared to *Ceratitis capitata* and *Drosophila melanogaster*. High throughput analysis of bacteria population of the *C. capitata* revealed only 5–23 OTUs and 7–13 OTU of bacteria symbionts to be associated with the adult fly and larva respectively at 97% sequence identity^21^. Similarly 454 pyrosequencing of gut bacteria community associated with *D. melanogaster* yielded a total of 122 OTU at 97% sequencing identity^22^.

The diversity of bacteria occupying *B. dorsalis* varied across different life stages of the fly. The bacteria diversity was similar across immature stages (eggs and larvae), but greatly differed from the pupal and adult stages (Fig. 1, 2 & 3). These variations may have been influenced by their habitat and the type of diet these life stages were exposed to. Previous studies with mosquitoes have shown that bacteria diversity associated with mosquitoes living in different habitats were different^23^. Similarly host diet has been known to influence gut microbial diversity^24^,^25^. Previously *B. dorsalis* has been shown to have variation in gut bacterial diversity when adult flies were fed with full diet versus sugar diet^26^. Our study presents Proteobacteria as the most abundant phylum in the developmental stages, and Firmicutes as the most abundant phylum in the adult stages. This switch of the most abundant bacteria group from Proteobacteria to Firmicutes may be as a result of change in habitat and diet. A previous study has reported a correlation between *Enterococcus faecalis* (Firmicutes) and the host insect's ability to consume food^27^. In addition to the diet and habitat, the transmission patterns of different bacteria species may affect its presence in the different life stages. Members of the family *Enterobacteriaceae* have been reported to be vertically transmitted^6^,^28^, hence they are present in all life stages of the insect host.

Most of the abundant bacteria species found to be associated with *B. dorsalis* were either uncultured or found to be related to the bacteria present in the gut of *B. correcta*. It was interesting to realize that closely related congeneric flies^29^ share similar bacteria species. It is likely that many of these bacteria are specialized gut bacteria of *B. dorsalis* based on their similarity to bacterial strains in associated with *B. correcta*. These are likely to be the bacteria that could be useful targets for control measures. Future work looking at the infectious patterns of these bacteria among individual insects and modes of transmission would be the next step in understanding the importance of these particular bacteria and perhaps their vulnerability to manipulation.

Previously, most studies reported *Enterobacteriaceae* (Proteobacteria) as the most dominant bacteria family associated with tephritid flies^10^,^26^,^28^,^30^,^31^,^32^. Contrarily, our study showed the family *Enterococcaceae* (Firmicutes) to be the most dominant taxon in all life stages of *B. dorsalis* except the pupae. *Enterococcus* has previously been reported to be the most dominant genus associated with insecticide resistant stem borer strains^33^. This family was predominantly represented by one species related to *Enterococcus haemoperoxidus*, a gram positive bacteria of the genus *Enteroccoci* commonly isolated from human clinical samples^34^. *E. haemoperoxidus* has been reported to produce antibacterial substances with inhibitory activity against 21 G^+^ indicators^35^. Speculatively, it is possible that their presence in the gut of the oriental fruit fly is helping the fly to boost its immune system. Apart from *Enterococcus*, *Lactococcus* also made up a significant part of the Firmicutes. The *Drosophila* gut has also been shown to harbour large numbers of *Lactococcus*^22^.

The second most important bacteria associated with the oriental fruit fly was represented by uncultured environmental bacteria related to the genus *Comamonas* and belonging to the Proteobacteria Phylum. This genus made up a significant amount in all developmental stages representing the most abundant genus in the pupal stage but completely absent in the adults. Some members of this genus have been associated with the production of catalases^36^,^37^, which break down hydrogen peroxide to oxygen and water. This group in the immature stages may be helping the insects to cope with oxidative stress by supplementing available oxygen. However more studies need to be carried out to test this hypothesis.

An important family that has been previously reported to be associated with most fruit flies in the past is *Enterobacteriaceae*. Members of this group have been shown to play very important roles in courtship and reproduction. *Klebsiella oxytoca* has been shown to improve on the mating competitiveness in the medfly^38^. *Klebsiella pneumonia*, *Citrobacter feundii* and *Enterobacter cloacae* have been shown to act as attractive lures for Tephritidae^10^,^39^,^40^,^41^. Low levels recorded at the adult gut may be as a result of relocation of these bacteria to the reproductive system where it is abundant^41^. However it is possible that apart from reproduction, this group might be playing other important roles in the developmental stages.

## Conclusion

This work has enabled a deeper understanding of the bacterial symbionts associated with different life stages of *B. dorsalis*. Some important symbionts that were previously not known to be associated with the oriental fruit fly have been brought to light. These are the members of the family *Comamonadaceae* and *Enterococcaceae*. The gut of *B. dorsalis* harbors a large diversity of symbiotic bacteria representing six phyla. These bacteria varied across the developmental stages. *Enterococcaceae* was the most abundant family in the adult insects and *Comamonadaceae* was the most abundant family in the immature stages. This study provides new clues on symbiotic bacteria that could be exploited in *B. dorsalis* bio-control programs. These involve some unique reads that were similar to those found in the gut of *B. correcta*, which may be potentially important for the biology of this fly. Understanding the specific functions and the transmission patterns of these species will be a fertile area for future research.

## Methods

### Sample collection

Insects were collected from Huazhong Agricultural University, Wuhan, China (30°4′ N and 114°3′ E). Methyl eugenol (4-Allyl-1, 2-dimethoxybenzene) traps and protein baits were used to collect the adults of *B. dorsalis*. Eggs and first instar larva were collected from healthy fruits that had oviposition sites by carefully removing the peel and examining the peel pulp interface and the pulp for the presence of viable insects. Third instar larva were collected from fallen fruits. The pupae were collected by allowing matured third instar larva to pupate in sterile sand in the laboratory. After sampling, insects were transported to the laboratory and live insects were starved for at least 12 hours in order to clear the insect gut of allochthonous species before gut dissection.

### Insect Dissection and DNA extraction

Prior to dissection, ≈50 insects of each stage were washed in 70% ethanol, followed by 3 rinses in sterile distilled water. The experimental samples included the whole gut of the adult and third instar larva (from proventriculus to rectum, excluding Malpighian tubules), the whole first instar larva, pupae (without the puparium) and the eggs. To prevent contamination of the samples by bacteria from the surface of the insect^22^,^42^, gut dissection was performed in sterile distilled water on a sterilized glass slide with a pair of sterile tweezers under a stereomicroscope in a laminar flow hood. Total genomic DNA was extracted as follows; Insect samples were homogenized in Phosphate buffer (PBS), centrifuged and re-washed in the same buffer. Samples harvested by centrifugation were resuspended in 557 μl TE buffer. This was followed by incubation with 10 μl lysozyme (5 mg/ml) at 37°C for 20 min. Then 3 μl proteinaes K (20 mg/ml) and 30 μl SDS (10%) were added and incubated at 37°C for about 40 min. Finally 100 μl of NaCl (5 M) and 80 μl of CTAB/Nacl was added and the solution incubated at 65°C for 10 minutes. DNA extraction was carried out using phenol-chloroform-isoamyl alcohol mixed in the ratio 24:24:1 and centrifuged at 13,400 g for 4 min. Isopropyl alcohol was used to precipitate nucleic acids from the supernatant. Pellets were washed in 70% frozen alcohol and re-suspended in 30 μl of TE buffer.

### PCR amplification, amplicon quantification, pooling and Pyrosequencing

The variable region V1-3 of the 16s rRNA gene was selected for the construction of a bacterial community library through tag-Pyrosequencing. Bar coded broadly conserved primers 27F_5′ CCTATCCCCTGTGTGCCTTGGCAGTCTCAGAGAGTTTGATCCTGGCTCAG-3′, and 533R_5′-CCATCTCATCCCTGCGTGTCTCCGACGACTNNNNNNNNTTACCGCGGCTGCT GCAC -3′, containing the A and B sequencing adaptors (454 Life Sciences) were used for PCR to amplify ~536 bp region of the mentioned gene. The underlined portions in primer sequences represent the sequence of the A-adaptor while the “Ns” indicate the eight-base specific barcode sequence.

PCRs was carried out in 15 μL reactions in triplicate, with each reaction tube containing 0.2 mM of each primer, ~5 ng of template DNA, 1 × PCR reaction buffer, 1 U of *Pfu* DNA Polymerase (MBI. Fermentas, USA). The following condition was used for the PCR reactions: 94°C for 30 sec, 55°C for 30 sec and 72°C for 1 min for 25 cycles and a final extension of 72°C for 10 min. PCR products were subsequently subjected to electrophoresis on a 1.2% agarose gel, stained with ethidium bromide, and the targeted fragment size (~500 bp) purified with a DNA gel extraction kit (Axygen, China).

Prior to pyrosequencing, the concentration of the purified PCR product was checked and quality controlled using Quant-iT Pico Green double-stranded DNA assay (Invitrogen, Germany) and an Agilent 2100 bioanalyzer (Agilent, USA) respectively. After quantification, equimolar ratio from each mixture were pooled and subjected to emulsion PCR so as to generate amplicon libraries. A 454/Roche A sequencing primer kit on a Roche Genome Sequencer GS FLX Titanium platform was used to perform amplicon pyrosequencing from the A-end. This was carried out at National Human Genome Center at Shanghai, China.

### Statistical and bioinformatics analysis

Data preprocessing, OTU-based analysis, and hypothesis testing were performed on Mothur using the standard pipeline described at www.mothur.org/wiki/454_SOP, accessed 01 July, 2014^18^. Sff files were trimmed based on sequence quality using the shhh.flows script, and sequences with 1) any mismatches in the barcode, 2) more than two primer mismatches, 3) homopolymers of more than 8 bases, or 4) less than 200 bp were discarded. Unique sequences were then aligned using the silva reference alignment, and sequences within 1–2 bp of a more abundant sequence were pre-clustered together. Chimeras were identified and removed using uchime, and remaining unique sequences (731) were clustered into 172 OTU at 97% similarity^43^. Taxonomic classification of each OTU was done using RDP training set, version 9. OTU sequences that had at least 1% overall abundance (12 OTUs) were additionally blasted against the NCBI nucleotide collection (nr/nt) using the megablast algorithm and Green genes16S database to gain additional insight into taxonomic identity.

Bacterial community analyses were also conducted using Mothur^18^. To account for inequalities in sequence read depth among the samples, random subsamples of sequences were generated from each sample, equivalent to the number of sequences in the sample with the lowest coverage (5,967 sequences for the pupal sample). All community analyses were conducted on these reduced data, including ACE and Chao1 estimators of alpha diversity, Simpson and Shannon diversity indices, rarefaction, heatmaps of relative abundance, principal coordinate anaylsis (PCoA) and Good's estimate of coverage. The heatmap was generated using custom Perl scripts in line with the OTU distribution and abundance classification. Principal coordinate analysis were generated using Bayesian algorithms and dendrograms showing the similarity of bacterial communities in different life stages was constructed through jackknife-beta-diversity script from qiime.

## Supplementary Material

Supplementary Information Supplementary Information

## Figures and Tables

**Table 1:** Richness and diversity estimation of the 16S rRNA gene libraries from the pyrosequencing analysis

Sample	Cutoffs	OTUs	Ace	Chao	Shannon	Simpson	Coverage
BD1L	0.03	77	167.64	126.60	2.20	0.18	1.00
BD3L	0.03	81	103.24	97.87	2.45	0.12	1.00
BDE	0.03	76	146.87	114.75	2.20	0.19	1.00
BDF	0.03	59	85.75	87.11	1.56	0.33	1.00
BDM	0.03	54	252.54	129.60	0.97	0.63	1.00
BDP	0.03	60	171.54	132.50	1.52	0.35	0.99

**Table 2:** Abundance of 16S rRNA gene amplicons across developmental stages of *Bactrocera dorsalis*, expressed as % of total in each life stage

Order	Family	Bestmatch Genbank#	Bestmatch Genbank %	Best match Genbank ID	BDE	BD1L	BD3L	BDP	BDF	BDM
Lactobacillales	Enterococcaceae	JQ950506	97%	Bacterium from *B. correcta*	36.5	36.0	18.9	0.1	51.3	79.1
Burkholderiales	Comamonadaceae	JF692613	98%	Uncultured enviromental bacterium	12.8	11.0	18.8	53.9	0.0	0.0
unclassified	Unclassified	JQ950479	99%	Bacterium from *B. correcta*	12.0	14.1	12.6	0.1	19.4	2.7
Desulfovibrionales	Unclassified	JQ950461	98%	Bacterium from *B. correcta*	8.8	10.2	9.8	0.0	15.2	6.0
Enterobacteriales	Enterobacteriaceae	FJ393309.1	99.62%	*Enterobacter aerogenes*	9.9	9.6	14.5	21.6	0.7	0.3
Lactobacillales	Streptococcaceae	AB828413	98%	Uncultured environmental Bacterium	3.3	2.9	2.9	0.0	3.3	4.4
Burkholderiales	Comamonadaceae	KC853140	98%	Comamonas spp	3.1	2.8	5.3	8.4	0.0	0.0
Flavobacteriales	Flavobacteriaceae	JQ950512	98%	Bacterium from *B. correcta*	2.9	2.4	3.2	0.0	3.4	1.9
Burkholderiales	Comamonadaceae	KF785066	99%	Uncultured environmental bacterium	1.9	1.9	2.9	6.5	0.0	0.0
Bacteroidales	Porphyromonadaceae	JN680576	96%	Coackroach gut bacterium	1.1	1.2	1.1	0.0	1.6	2.0
Bacteroidales	Porphyromonadaceae	JX457996	93%	Coackroach gut bacterium	1.3	1.5	0.8	0.0	1.6	0.9
Pseudomonadales	Pseudomonadaceae	GU769300	98%	Bacterium from *B. correcta*	1.0	0.8	1.8	2.9	0.0	0.0
Pseudomonadales	Moraxellaceae	KC853125	100%	Bacterium from Frog	1.5	1.1	2.0	1.5	0.0	0.0
Others					4.0	4.5	5.3	4.9	3.4	2.7

*All data refer to gut samples isolated from the different life stages of *B. dorsalis*, apart from eggs and pupae.

*Only sequence reads that had at least a1% abundance in a life stage are presented.
