# Supplementary material for: Pyrosequencing reveals a shift in symbiotic bacteria populations across life stages of Bactrocera dorsalis
Source: Sci Rep. 2015 Mar 30;5:9470. doi: 10.1038/srep09470 (PMC5380164; doi:10.1038/srep09470)
Supplement: Supplementary Information [file srep09470-s1.pdf]

Pyrosequencing reveals a shift in symbiotic bacteria populations across life stages of *Bactrocera dorsalis*

Awawing A. Andongma[1], Lun Wan[1], Yong-Cheng Dong[1], Ping li[2], Nicolas Desneux[3], Jennifer A. White[4], Chang-Ying Niu[1*]

SUPPLEMENTARY INFORMATION

Supplementary sheet 1 (S1): Bacteria Taxonomy and abundance in gut of different developmental stages of *Bactrocera dorsalis*

OTU	totalseq	BDE	BD1L	BD3L	BDP	BDF	BDM	kingdom	phylum	class	order	family	genus
Otu001	18360	2723	2838	1441	6	4420	6932	Bacteria(100)	Firmicutes(100)	Bacilli(100)	Lactobacillales(100)	Enterococcaceae(100)	unclassified(100)
Otu002	6472	954	867	1437	3214	0	0	Bacteria(100)	Proteobacteria(100)	Betaproteobacteria(100)	Burkholderiales(100)	Comamonadaceae(100)	Comamonas(100)
Otu003	4890	897	1116	962	7	1671	237	Bacteria(100)	Proteobacteria(100)	Gammaproteobacteria(100)	unclassified(100)	unclassified(100)	unclassified(100)
Otu004	4048	655	807	745	1	1313	527	Bacteria(100)	Proteobacteria(100)	Deltaproteobacteria(100)	Desulfovibrionales(94)	unclassified(94)	unclassified(94)
Otu005	3977	737	759	1110	1286	63	22	Bacteria(100)	Proteobacteria(100)	Gammaproteobacteria(100)	Enterobacteriales(100)	Enterobacteriaceae(100)	unclassified(91)
Otu006	1367	245	230	218	0	285	389	Bacteria(100)	Firmicutes(100)	Bacilli(100)	Lactobacillales(100)	Streptococcaceae(100)	Lactococcus(100)
Otu007	1353	232	219	402	500	0	0	Bacteria(100)	Proteobacteria(100)	Betaproteobacteria(100)	Burkholderiales(100)	Comamonadaceae(100)	unclassified(100)
Otu008	1109	216	189	247	2	291	164	Bacteria(100)	Bacteroidetes(100)	Flavobacteria(100)	Flavobacteriales(100)	Flavobacteriaceae(100)	unclassified(100)
Otu009	902	143	149	220	390	0	0	Bacteria(100)	Proteobacteria(100)	Betaproteobacteria(100)	Burkholderiales(100)	Comamonadaceae(100)	Comamonas(100)
Otu010	580	81	93	87	0	141	178	Bacteria(100)	Bacteroidetes(100)	Bacteroidia(100)	Bacteroidales(100)	Porphyromonadaceae(100)	unclassified(100)
Otu011	491	96	117	64	2	134	78	Bacteria(100)	Bacteroidetes(100)	Bacteroidia(100)	Bacteroidales(100)	Porphyromonadaceae(100)	unclassified(100)
Otu012	453	76	63	138	176	0	0	Bacteria(100)	Proteobacteria(100)	Gammaproteobacteria(100)	Pseudomonadales(100)	Pseudomonadaceae(100)	Pseudomonas(99)
Otu013	444	110	89	154	91	0	0	Bacteria(100)	Proteobacteria(100)	Gammaproteobacteria(100)	Pseudomonadales(100)	Moraxellaceae(100)	Acinetobacter(100)
Otu014	352	66	71	60	1	83	71	Bacteria(100)	Bacteroidetes(100)	Bacteroidia(100)	Bacteroidales(100)	Porphyromonadaceae(100)	Dysgonomonas(100)
Otu015	206	44	42	48	1	37	34	Bacteria(100)	Firmicutes(100)	Bacilli(100)	Lactobacillales(100)	unclassified(100)	unclassified(100)
Otu016	159	21	14	21	103	0	0	Bacteria(100)	Proteobacteria(100)	Alphaproteobacteria(100)	Rhodospirillales(100)	Acetobacteraceae(100)	Acetobacter(74)
Otu017	114	24	21	40	29	0	0	Bacteria(100)	Firmicutes(100)	Bacilli(100)	Lactobacillales(100)	Lactobacillaceae(94)	Lactobacillus(94)
Otu018	98	12	28	11	0	35	12	Bacteria(100)	Proteobacteria(100)	Deltaproteobacteria(100)	Desulfovibrionales(89)	unclassified(89)	unclassified(89)

Otu019	61	8	16	22	15	0	0	Bacteria(100)	Firmicutes(100)	Bacilli(100)	Bacillales(100)	Staphylococcaceae(100)	Staphylococcus(100)
Otu020	50	8	13	10	0	18	1	Bacteria(100)	Proteobacteria(100)	Gammaproteobacteria(100)	Gammaproteobacteria_order_incertae_sedis(92)	Gammaproteobacteria_family_incertae_sedis(92)	Orbus(92)
Otu021	48	7	11	9	0	7	14	Bacteria(100)	Firmicutes(100)	Bacilli(100)	Lactobacillales(100)	Lactobacillaceae(100)	Lactobacillus(75)
Otu022	43	7	6	15	15	0	0	Bacteria(100)	Proteobacteria(100)	Gammaproteobacteria(100)	Xanthomonadales(100)	Xanthomonadaceae(100)	unclassified(100)
Otu023	41	5	7	13	16	0	0	Bacteria(100)	Firmicutes(100)	Bacilli(100)	Lactobacillales(100)	Lactobacillaceae(98)	Lactobacillus(98)
Otu024	35	6	7	4	3	6	9	Bacteria(100)	Firmicutes(100)	Bacilli(100)	Lactobacillales(100)	Enterococcaceae(100)	Enterococcus(100)
Otu025	35	4	12	4	0	3	12	Bacteria(100)	Firmicutes(100)	unclassified(100)	unclassified(100)	unclassified(100)	unclassified(100)
Otu026	33	3	9	5	0	6	10	Bacteria(100)	Firmicutes(100)	Bacilli(100)	Lactobacillales(100)	Streptococcaceae(100)	Lactococcus(100)
Otu027	31	6	7	6	6	6	0	Bacteria(100)	Actinobacteria(100)	Actinobacteria(100)	Actinomycetales(100)	Propionibacteriaceae(100)	Propionibacterium(100)
Otu028	27	4	4	7	12	0	0	Bacteria(100)	Firmicutes(100)	Bacilli(100)	Bacillales(100)	Bacillales_Incertae_Sedis_XII(100)	Exiguobacterium(100)
Otu029	26	4	4	2	0	7	9	Bacteria(100)	Bacteroidetes(100)	Bacteroidia(100)	Bacteroidales(100)	Porphyromonadaceae(100)	Dysgonomonas(100)
Otu030	26	6	6	7	7	0	0	Bacteria(100)	Proteobacteria(100)	Gammaproteobacteria(100)	Pseudomonadales(100)	Moraxellaceae(100)	Acinetobacter(100)
Otu031	24	3	5	4	6	4	2	Bacteria(100)	Proteobacteria(100)	Gammaproteobacteria(100)	Enterobacteriales(100)	Enterobacteriaceae(100)	unclassified(100)
Otu032	23	0	5	8	0	5	5	Bacteria(100)	Proteobacteria(100)	Gammaproteobacteria(100)	unclassified(100)	unclassified(100)	unclassified(100)
Otu033	22	1	5	1	15	0	0	Bacteria(100)	Proteobacteria(100)	Betaproteobacteria(96)	Burkholderiales(96)	Comamonadaceae(96)	unclassified(96)
Otu034	21	2	2	4	0	4	9	Bacteria(100)	Firmicutes(100)	Bacilli(100)	Lactobacillales(100)	Enterococcaceae(100)	Vagococcus(100)
Otu035	21	1	2	5	0	11	2	Bacteria(100)	Proteobacteria(100)	Gammaproteobacteria(100)	Enterobacteriales(100)	Enterobacteriaceae(100)	Escherichia_Shigella(96)
Otu036	16	2	0	12	0	2	0	Bacteria(100)	Proteobacteria(100)	Gammaproteobacteria(100)	Gammaproteobacteria_order_incertae_sedis(88)	Gammaproteobacteria_family_incertae_sedis(88)	Orbus(88)
Otu037	11	5	2	2	0	0	2	Bacteria(100)	Bacteroidetes(100)	Bacteroidia(100)	Bacteroidales(100)	Porphyromonadaceae(100)	Dysgonomonas(55)
Otu038	11	2	2	0	0	1	6	Bacteria(100)	Firmicutes(100)	Bacilli(100)	Lactobacillales(100)	Enterococcaceae(100)	Enterococcus(82)
Otu039	11	0	2	6	3	0	0	Bacteria(100)	Firmicutes(100)	Bacilli(100)	Bacillales(100)	unclassified(82)	unclassified(82)
Otu040	10	1	0	1	8	0	0	Bacteria(100)	Proteobacteria(100)	Alphaproteobacteria(100)	Sphingomonadales(100)	Sphingomonadaceae(100)	Sphingomonas(100)
Otu041	10	2	2	4	0	1	1	Bacteria(100)	Proteobacteria(100)	Gammaproteobacteria(100)	unclassified(100)	unclassified(100)	unclassified(100)

Otu042	10	0	5	2	0	2	1	Bacteria(100)	Proteobacteria(100)	Gammaproteobacteria(100)	Enterobacteriales(100)	Enterobacteriaceae(100)	Morganella(100)
Otu043	8	2	1	4	1	0	0	Bacteria(100)	Firmicutes(100)	Bacilli(100)	Bacillales(100)	Bacillales_Incertae_Sedis_XII(100)	Exiguobacterium(100)
Otu044	8	1	1	2	0	4	0	Bacteria(100)	Proteobacteria(100)	Gammaproteobacteria(100)	unclassified(88)	unclassified(88)	unclassified(88)
Otu045	8	0	0	4	3	1	0	Bacteria(100)	Firmicutes(100)	Bacilli(100)	Bacillales(100)	Staphylococcaceae(100)	Staphylococcus(100)
Otu046	8	0	4	0	0	3	1	Bacteria(100)	Proteobacteria(100)	Gammaproteobacteria(100)	Enterobacteriales(100)	Enterobacteriaceae(100)	unclassified(100)
Otu047	8	0	2	1	5	0	0	Bacteria(100)	Proteobacteria(100)	Gammaproteobacteria(100)	Enterobacteriales(100)	Enterobacteriaceae(100)	Klebsiella(63)
Otu048	8	2	1	3	2	0	0	Bacteria(100)	Proteobacteria(100)	Alphaproteobacteria(100)	Rhizobiales(100)	Rhizobiaceae(88)	Rhizobium(88)
Otu049	7	0	1	0	6	0	0	Bacteria(100)	Proteobacteria(100)	Gammaproteobacteria(100)	Enterobacteriales(100)	Enterobacteriaceae(100)	unclassified(86)
Otu050	7	2	4	0	0	0	1	Bacteria(100)	Firmicutes(100)	Erysipelotrichia(100)	Erysipelotrichales(100)	Erysipelotrichaceae(100)	Erysipelothrix(100)
Otu051	6	0	1	4	1	0	0	Bacteria(100)	Proteobacteria(100)	Gammaproteobacteria(100)	Pseudomonadales(100)	Moraxellaceae(100)	Acinetobacter(100)
Otu052	6	1	0	2	0	3	0	Bacteria(100)	Firmicutes(100)	Bacilli(100)	Lactobacillales(100)	Streptococcaceae(100)	Lactococcus(100)
Otu053	6	1	0	0	5	0	0	Bacteria(100)	Proteobacteria(100)	Betaproteobacteria(100)	Burkholderiales(100)	Comamonadaceae(100)	unclassified(100)
Otu054	6	0	0	4	1	1	0	Bacteria(100)	Firmicutes(100)	Bacilli(100)	Lactobacillales(100)	Streptococcaceae(100)	Streptococcus(100)
Otu055	6	1	0	0	0	4	1	Bacteria(100)	Firmicutes(100)	Bacilli(100)	Lactobacillales(100)	unclassified(100)	unclassified(100)
Otu056	6	2	0	1	0	0	3	Bacteria(100)	Firmicutes(100)	Bacilli(100)	Lactobacillales(100)	Leuconostocaceae(100)	Leuconostoc(100)
Otu057	6	0	0	2	0	3	1	Bacteria(100)	Proteobacteria(100)	Gammaproteobacteria(100)	Enterobacteriales(100)	Enterobacteriaceae(100)	unclassified(100)
Otu058	6	0	1	4	1	0	0	Bacteria(100)	Actinobacteria(100)	Actinobacteria(100)	Actinomycetales(100)	Microbacteriaceae(100)	Microbacterium(67)
Otu059	5	1	0	2	2	0	0	Bacteria(100)	Proteobacteria(100)	Betaproteobacteria(100)	Burkholderiales(100)	Comamonadaceae(100)	unclassified(100)
Otu060	5	0	1	2	0	1	1	Bacteria(100)	Proteobacteria(100)	Gammaproteobacteria(100)	unclassified(100)	unclassified(100)	unclassified(100)
Otu061	5	2	0	2	0	0	1	Bacteria(100)	Proteobacteria(100)	Gammaproteobacteria(100)	unclassified(100)	unclassified(100)	unclassified(100)
Otu062	5	1	2	1	1	0	0	Bacteria(100)	Proteobacteria(100)	Gammaproteobacteria(100)	Pseudomonadales(100)	Pseudomonadaceae(100)	Pseudomonas(100)
Otu063	5	0	0	5	0	0	0	Bacteria(100)	Firmicutes(100)	Bacilli(100)	Lactobacillales(100)	unclassified(100)	unclassified(100)
Otu064	5	0	0	0	0	5	0	Bacteria(100)	Bacteroidetes(100)	Bacteroidia(100)	Bacteroidales(100)	Porphyromonadaceae(100)	Dysgonomonas(100)
Otu065	5	3	1	0	1	0	0	Bacteria(100)	Firmicutes(100)	Bacilli(100)	Lactobacillales(100)	Streptococcaceae(100)	Streptococcus(100)
Otu066	4	0	1	1	0	1	1	Bacteria(100)	Firmicutes(100)	Bacilli(100)	Lactobacillales(100)	Leuconostocaceae(100)	Fructobacillus(100)

Otu067	4	1	0	2	0	0	1	Bacteria(100)	Bacteroidetes(100)	Bacteroidia(100)	Bacteroidales(100)	Porphyromonadaceae(100)	unclassified(100)
Otu068	4	0	0	1	0	0	3	Bacteria(100)	Bacteroidetes(100)	Bacteroidia(100)	Bacteroidales(100)	Porphyromonadaceae(100)	Dysgonomonas(100)
Otu069	4	1	0	0	0	2	1	Bacteria(100)	Bacteroidetes(100)	Bacteroidia(100)	Bacteroidales(100)	Porphyromonadaceae(100)	Dysgonomonas(100)
Otu070	4	3	0	1	0	0	0	Bacteria(100)	Proteobacteria(100)	Gammaproteobacteria(100)	unclassified(100)	unclassified(100)	unclassified(100)
Otu071	4	0	0	0	0	1	3	Bacteria(100)	Firmicutes(100)	Erysipelotrichia(100)	Erysipelotrichales(100)	Erysipelotrichaceae(100)	Erysipelothrix(100)
Otu072	4	1	0	2	0	1	0	Bacteria(100)	Proteobacteria(100)	Gammaproteobacteria(100)	Xanthomonadales(100)	Xanthomonadaceae(100)	Stenotrophomonas(100)
Otu073	3	1	0	0	2	0	0	Bacteria(100)	Proteobacteria(100)	Betaproteobacteria(100)	Burkholderiales(100)	Comamonadaceae(100)	unclassified(100)
Otu074	3	0	0	3	0	0	0	Bacteria(100)	Firmicutes(100)	Bacilli(100)	Bacillales(100)	Bacillaceae_1(100)	Bacillus(100)
Otu075	3	1	1	0	0	0	1	Bacteria(100)	unclassified(100)	unclassified(100)	unclassified(100)	unclassified(100)	unclassified(100)
Otu076	3	0	1	0	0	1	1	Bacteria(100)	Firmicutes(100)	Bacilli(100)	Lactobacillales(100)	unclassified(67)	unclassified(67)
Otu077	3	0	0	0	1	2	0	Bacteria(100)	Actinobacteria(100)	Actinobacteria(100)	Actinomycetales(100)	Corynebacteriaceae(100)	Corynebacterium(100)
Otu078	3	0	0	1	0	0	2	Bacteria(100)	Firmicutes(100)	Bacilli(100)	Lactobacillales(100)	unclassified(100)	unclassified(100)
Otu079	3	0	0	1	0	2	0	Bacteria(100)	Bacteroidetes(100)	Bacteroidia(100)	Bacteroidales(100)	Porphyromonadaceae(100)	Dysgonomonas(100)
Otu080	3	2	0	0	0	1	0	Bacteria(100)	Proteobacteria(100)	Gammaproteobacteria(100)	unclassified(67)	unclassified(67)	unclassified(67)
Otu081	3	0	0	2	1	0	0	Bacteria(100)	Actinobacteria(100)	Actinobacteria(100)	Actinomycetales(100)	unclassified(67)	unclassified(67)
Otu082	3	0	0	1	0	2	0	Bacteria(100)	Bacteroidetes(100)	Bacteroidia(100)	Bacteroidales(100)	Bacteroidaceae(100)	Bacteroides(100)
Otu083	3	0	2	0	1	0	0	Bacteria(100)	Proteobacteria(100)	Betaproteobacteria(100)	Burkholderiales(100)	Comamonadaceae(100)	unclassified(100)
Otu084	2	2	0	0	0	0	0	Bacteria(100)	Proteobacteria(100)	Gammaproteobacteria(100)	Enterobacteriales(100)	Enterobacteriaceae(100)	unclassified(100)
Otu085	2	0	1	0	1	0	0	Bacteria(100)	Proteobacteria(100)	Betaproteobacteria(100)	Burkholderiales(100)	Comamonadaceae(100)	unclassified(100)
Otu086	2	1	0	0	1	0	0	Bacteria(100)	Bacteroidetes(100)	Flavobacteria(100)	Flavobacteriales(100)	Flavobacteriaceae(100)	Chryseobacterium(100)
Otu087	2	1	0	0	0	0	1	Bacteria(100)	Bacteroidetes(100)	Bacteroidia(100)	Bacteroidales(100)	Porphyromonadaceae(100)	Dysgonomonas(100)
Otu088	2	0	0	2	0	0	0	Bacteria(100)	Proteobacteria(100)	Betaproteobacteria(100)	Burkholderiales(100)	Comamonadaceae(100)	unclassified(100)
Otu089	2	0	0	1	0	1	0	Bacteria(100)	Proteobacteria(100)	Gammaproteobacteria(100)	unclassified(100)	unclassified(100)	unclassified(100)
Otu090	2	0	0	0	0	2	0	Bacteria(100)	Proteobacteria(100)	Gammaproteobacteria(100)	Enterobacteriales(100)	Enterobacteriaceae(100)	unclassified(100)
Otu091	2	1	1	0	0	0	0	Bacteria(100)	Bacteroidetes(100)	Bacteroidia(100)	Bacteroidales(100)	Porphyromonadaceae(100)	unclassified
Otu092	2	0	0	0	0	2	0	Bacteria(100)	Firmicutes(100)	Bacilli(100)	Lactobacillales(100)	unclassified(100)	unclassified(100)

Otu093	2	1	1	0	0	0	0	Bacteria(100)	Proteobacteria(100)	Gammaproteobacteria(100)	Enterobacteriales(100)	Enterobacteriaceae(100)	unclassified(100)
Otu094	2	1	0	0	0	1	0	Bacteria(100)	unclassified	unclassified	unclassified	unclassified	unclassified
Otu095	2	0	0	2	0	0	0	Bacteria(100)	Proteobacteria(100)	unclassified(100)	unclassified(100)	unclassified(100)	unclassified(100)
Otu096	2	1	1	0	0	0	0	Bacteria(100)	Proteobacteria(100)	Gammaproteobacteria(100)	unclassified(100)	unclassified(100)	unclassified(100)
Otu097	2	0	0	1	0	1	0	Bacteria(100)	Proteobacteria(100)	Gammaproteobacteria(100)	Enterobacteriales(100)	Enterobacteriaceae(100)	unclassified(100)
Otu098	2	1	0	1	0	0	0	Bacteria(100)	Bacteroidetes(100)	Bacteroidia(100)	Bacteroidales(100)	Porphyromonadaceae(100)	Dysgonomonas(100)
Otu099	2	1	0	0	0	0	1	Bacteria(100)	Bacteroidetes(100)	Bacteroidia(100)	Bacteroidales(100)	Porphyromonadaceae(100)	Dysgonomonas(100)
Otu100	1	0	0	0	0	0	1	Bacteria(100)	Bacteroidetes(100)	Bacteroidia(100)	Bacteroidales(100)	Porphyromonadaceae(100)	Dysgonomonas(100)
Otu101	1	0	0	0	0	0	1	Bacteria(100)	Firmicutes(100)	Bacilli(100)	Lactobacillales(100)	unclassified(100)	unclassified(100)
Otu102	1	1	0	0	0	0	0	Bacteria(100)	Bacteroidetes(100)	Sphingobacteria(100)	Sphingobacteriales(100)	Sphingobacteriaceae(100)	Sphingobacterium(100)
Otu103	1	0	0	0	1	0	0	Bacteria(100)	Proteobacteria(100)	Betaproteobacteria(100)	Burkholderiales(100)	Comamonadaceae(100)	Comamonas(100)
Otu104	1	0	0	1	0	0	0	Bacteria(100)	Proteobacteria(100)	Gammaproteobacteria(100)	Enterobacteriales(100)	Enterobacteriaceae(100)	unclassified(100)
Otu105	1	0	0	0	0	0	1	Bacteria(100)	Firmicutes(100)	Bacilli(100)	Lactobacillales(100)	Carnobacteriaceae(100)	Granulicatella(100)
Otu106	1	0	0	0	0	0	1	Bacteria(100)	Firmicutes(100)	Bacilli(100)	Lactobacillales(100)	Lactobacillaceae(100)	Lactobacillus(100)
Otu107	1	0	0	1	0	0	0	Bacteria(100)	Proteobacteria(100)	Alphaproteobacteria(100)	Caulobacterales(100)	Caulobacteraceae(100)	Brevundimonas(100)
Otu108	1	0	0	0	1	0	0	Bacteria(100)	Proteobacteria(100)	Alphaproteobacteria(100)	Caulobacterales(100)	Caulobacteraceae(100)	unclassified(100)
Otu109	1	1	0	0	0	0	0	Bacteria(100)	Bacteroidetes(100)	Bacteroidia(100)	Bacteroidales(100)	Porphyromonadaceae(100)	Dysgonomonas(100)
Otu110	1	0	0	0	1	0	0	Bacteria(100)	Firmicutes(100)	Bacilli(100)	Bacillales(100)	Bacillales_Incertae_Sedis_XI(100)	Gemella(100)
Otu111	1	0	0	1	0	0	0	Bacteria(100)	Deinococcus-Thermus(100)	Deinococci(100)	Thermales(100)	Thermaceae(100)	Meiothermus(100)
Otu112	1	0	0	0	0	0	1	Bacteria(100)	Bacteroidetes(100)	Bacteroidia(100)	Bacteroidales(100)	Porphyromonadaceae(100)	Dysgonomonas(100)
Otu113	1	0	0	0	1	0	0	Bacteria(100)	Bacteroidetes(100)	Sphingobacteria(100)	Sphingobacteriales(100)	Cytophagaceae(100)	Hymenobacter(100)
Otu114	1	0	1	0	0	0	0	Bacteria(100)	Proteobacteria(100)	Betaproteobacteria(100)	Burkholderiales(100)	Comamonadaceae(100)	unclassified(100)
Otu115	1	0	1	0	0	0	0	Bacteria(100)	Bacteroidetes(100)	Bacteroidia(100)	Bacteroidales(100)	Porphyromonadaceae(100)	unclassified(100)
Otu116	1	0	1	0	0	0	0	Bacteria(100)	Firmicutes(100)	Bacilli(100)	Lactobacillales(100)	unclassified(100)	unclassified(100)

Otu117	1	0	1	0	0	0	0	Bacteria(100)	Firmicutes(100)	Bacilli(100)	Lactobacillales(100)	Lactobacillaceae(100)	Lactobacillus(100)
Otu118	1	1	0	0	0	0	0	Bacteria(100)	Proteobacteria(100)	Gammaproteobacteria(100)	Gammaproteobacteria_order_incertae_sedis(100)	Gammaproteobacteria_family_incertae_sedis(100)	Orbus(100)
Otu119	1	0	0	0	1	0	0	Bacteria(100)	Proteobacteria(100)	Alphaproteobacteria(100)	Rhizobiales(100)	Brucellaceae(100)	unclassified(100)
Otu120	1	0	0	0	0	1	0	Bacteria(100)	Firmicutes(100)	Bacilli(100)	Lactobacillales(100)	Carnobacteriaceae(100)	Granulicatella(100)
Otu121	1	0	0	0	0	1	0	Bacteria(100)	Firmicutes(100)	Bacilli(100)	Lactobacillales(100)	unclassified(100)	unclassified(100)
Otu122	1	0	0	1	0	0	0	Bacteria(100)	Proteobacteria(100)	Gammaproteobacteria(100)	Pseudomonadales(100)	Moraxellaceae(100)	Acinetobacter(100)
Otu123	1	0	0	0	0	1	0	Bacteria(100)	Bacteroidetes(100)	Bacteroidia(100)	Bacteroidales(100)	Prevotellaceae(100)	Prevotella(100)
Otu124	1	0	0	1	0	0	0	Bacteria(100)	Proteobacteria(100)	Gammaproteobacteria(100)	Enterobacteriales(100)	Enterobacteriaceae(100)	unclassified(100)
Otu125	1	0	0	1	0	0	0	Bacteria(100)	Proteobacteria(100)	Gammaproteobacteria(100)	Enterobacteriales(100)	Enterobacteriaceae(100)	Klebsiella(100)
Otu126	1	0	0	0	0	1	0	Bacteria(100)	Proteobacteria(100)	Gammaproteobacteria(100)	Enterobacteriales(100)	Enterobacteriaceae(100)	Pantoea(100)
Otu127	1	0	0	0	0	1	0	Bacteria(100)	Proteobacteria(100)	Gammaproteobacteria(100)	Enterobacteriales(100)	Enterobacteriaceae(100)	unclassified(100)
Otu128	1	0	0	1	0	0	0	Bacteria(100)	Proteobacteria(100)	Alphaproteobacteria(100)	Rhizobiales(100)	Methylobacteriaceae(100)	Methylobacterium(100)
Otu129	1	0	0	0	0	1	0	Bacteria(100)	Bacteroidetes(100)	Bacteroidia(100)	Bacteroidales(100)	Porphyromonadaceae(100)	unclassified(100)
Otu130	1	0	0	0	0	1	0	Bacteria(100)	unclassified(100)	unclassified(100)	unclassified(100)	unclassified(100)	unclassified(100)
Otu131	1	0	0	1	0	0	0	Bacteria(100)	Firmicutes(100)	Bacilli(100)	Lactobacillales(100)	Enterococcaceae(100)	Enterococcus(100)
Otu132	1	0	0	0	0	1	0	Bacteria(100)	Bacteroidetes(100)	Bacteroidia(100)	Bacteroidales(100)	Porphyromonadaceae(100)	Dysgonomonas(100)
Otu133	1	0	0	0	0	0	1	Bacteria(100)	Proteobacteria(100)	Betaproteobacteria(100)	Burkholderiales(100)	Burkholderiaceae(100)	Ralstonia(100)
Otu134	1	0	0	0	1	0	0	Bacteria(100)	Proteobacteria(100)	Alphaproteobacteria(100)	Rhodospirillales(100)	Acetobacteraceae(100)	unclassified(100)
Otu135	1	0	0	0	0	1	0	Bacteria(100)	Firmicutes(100)	Bacilli(100)	Lactobacillales(100)	unclassified(100)	unclassified(100)
Otu136	1	0	0	0	0	1	0	Bacteria(100)	Firmicutes(100)	Bacilli(100)	Lactobacillales(100)	Streptococcaceae(100)	Lactococcus(100)
Otu137	1	0	0	1	0	0	0	Bacteria(100)	Deinococcus-Thermus(100)	Deinococci(100)	Thermales(100)	Thermaceae(100)	Thermus(100)
Otu138	1	0	0	0	1	0	0	Bacteria(100)	Proteobacteria(100)	Gammaproteobacteria(100)	Enterobacteriales(100)	Enterobacteriaceae(100)	unclassified(100)
Otu139	1	0	0	0	0	0	1	Bacteria(100)	Firmicutes(100)	Bacilli(100)	Lactobacillales(100)	Enterococcaceae(100)	unclassified(100)
Otu140	1	0	0	0	0	0	1	Bacteria(100)	Bacteroidetes(100)	Bacteroidia(100)	Bacteroidales(100)	Porphyromonadaceae(100)	Dysgonomonas(100)

Otu141	1	0	0	0	1	0	0	Bacteria(100)	Proteobacteria(100)	Betaproteobacteria(100)	Burkholderiales(100)	Comamonadaceae(100)	unclassified(100)
Otu142	1	0	0	0	0	0	1	Bacteria(100)	Proteobacteria(100)	Gammaproteobacteria(100)	Xanthomonadales(100)	Xanthomonadaceae(100)	unclassified(100)
Otu143	1	0	0	0	0	0	1	Bacteria(100)	Proteobacteria(100)	Betaproteobacteria(100)	Neisseriales(100)	Neisseriaceae(100)	Uruburuella(100)
Otu144	1	0	0	0	0	0	1	Bacteria(100)	Firmicutes(100)	Bacilli(100)	Lactobacillales(100)	Enterococcaceae(100)	unclassified(100)
Otu145	1	0	0	0	0	0	1	Bacteria(100)	Proteobacteria(100)	Gammaproteobacteria(100)	Xanthomonadales(100)	Xanthomonadaceae(100)	Wohlfahrtiimonas(100)
Otu146	1	0	0	0	1	0	0	Bacteria(100)	Proteobacteria(100)	Gammaproteobacteria(100)	Enterobacteriales(100)	Enterobacteriaceae(100)	unclassified(100)
Otu147	1	0	1	0	0	0	0	Bacteria(100)	Firmicutes(100)	Bacilli(100)	Lactobacillales(100)	Enterococcaceae(100)	unclassified(100)
Otu148	1	1	0	0	0	0	0	Bacteria(100)	Proteobacteria(100)	Gammaproteobacteria(100)	Xanthomonadales(100)	Xanthomonadaceae(100)	Rudaea(100)
Otu149	1	1	0	0	0	0	0	Bacteria(100)	Proteobacteria(100)	Betaproteobacteria(100)	Burkholderiales(100)	Burkholderiaceae(100)	Cupriavidus(100)
Otu150	1	0	1	0	0	0	0	Bacteria(100)	Bacteroidetes(100)	Flavobacteria(100)	Flavobacteriales(100)	Flavobacteriaceae(100)	unclassified(100)
Otu151	1	0	1	0	0	0	0	Bacteria(100)	Proteobacteria(100)	Gammaproteobacteria(100)	Enterobacteriales(100)	Enterobacteriaceae(100)	Klebsiella(100)
Otu152	1	0	0	0	1	0	0	Bacteria(100)	Proteobacteria(100)	Betaproteobacteria(100)	unclassified(100)	unclassified(100)	unclassified(100)
Otu153	1	1	0	0	0	0	0	Bacteria(100)	Proteobacteria(100)	Gammaproteobacteria(100)	Enterobacteriales(100)	Enterobacteriaceae(100)	unclassified(100)
Otu154	1	1	0	0	0	0	0	Bacteria(100)	unclassified(100)	unclassified(100)	unclassified(100)	unclassified(100)	unclassified(100)
Otu155	1	0	0	0	1	0	0	Bacteria(100)	Proteobacteria(100)	Gammaproteobacteria(100)	Enterobacteriales(100)	Enterobacteriaceae(100)	unclassified(100)
Otu156	1	0	1	0	0	0	0	Bacteria(100)	Fusobacteria(100)	Fusobacteria(100)	Fusobacteriales(100)	Fusobacteriaceae(100)	Fusobacterium(100)
Otu157	1	0	0	0	1	0	0	Bacteria(100)	Proteobacteria(100)	Alphaproteobacteria(100)	Rhodospirillales(100)	Acetobacteraceae(100)	unclassified(100)
Otu158	1	1	0	0	0	0	0	Bacteria(100)	Proteobacteria(100)	Gammaproteobacteria(100)	Enterobacteriales(100)	Enterobacteriaceae(100)	unclassified(100)
Otu159	1	0	0	0	1	0	0	Bacteria(100)	Proteobacteria(100)	Alphaproteobacteria(100)	Rhodospirillales(100)	Acetobacteraceae(100)	Acetobacter(100)
Otu160	1	0	0	0	1	0	0	Bacteria(100)	Proteobacteria(100)	Gammaproteobacteria(100)	unclassified(100)	unclassified(100)	unclassified(100)
Otu161	1	0	1	0	0	0	0	Bacteria(100)	Bacteroidetes(100)	Sphingobacteria(100)	Sphingobacteriales(100)	unclassified(100)	unclassified(100)
Otu162	1	0	1	0	0	0	0	Bacteria(100)	Firmicutes(100)	Bacilli(100)	Lactobacillales(100)	Enterococcaceae(100)	unclassified(100)
Otu163	1	0	1	0	0	0	0	Bacteria(100)	Proteobacteria(100)	Gammaproteobacteria(100)	Enterobacteriales(100)	Enterobacteriaceae(100)	unclassified(100)
Otu164	1	0	1	0	0	0	0	Bacteria(100)	Proteobacteria(100)	Gammaproteobacteria(100)	unclassified(100)	unclassified(100)	unclassified(100)
Otu165	1	0	0	0	1	0	0	Bacteria(100)	Proteobacteria(100)	Betaproteobacteria(100)	Neisseriales(100)	Neisseriaceae(100)	Neisseria(100)
Otu166	1	0	1	0	0	0	0	Bacteria(100)	Proteobacteria(100)	Gammaproteobacteria(100)	unclassified(100)	unclassified(100)	unclassified(100)

Otu167	1	0	1	0	0	0	0	Bacteria(100)	Proteobacteria(100)	Gammaproteobacteria(100)	unclassified(100)	unclassified(100)	unclassified(100)
Otu168	1	0	1	0	0	0	0	Bacteria(100)	Proteobacteria(100)	Gammaproteobacteria(100)	Xanthomonadales(100)	Xanthomonadaceae(100)	unclassified(100)
Otu169	1	0	1	0	0	0	0	Bacteria(100)	Proteobacteria(100)	Gammaproteobacteria(100)	Enterobacteriales(100)	Enterobacteriaceae(100)	unclassified(100)
Otu170	1	1	0	0	0	0	0	Bacteria(100)	Proteobacteria(100)	Betaproteobacteria(100)	Burkholderiales(100)	Comamonadaceae(100)	unclassified(100)
Otu171	1	0	1	0	0	0	0	Bacteria(100)	Proteobacteria(100)	Gammaproteobacteria(100)	Pseudomonadales(100)	Moraxellaceae(100)	Acinetobacter(100)
Otu172	1	0	0	0	1	0	0	Bacteria(100)	Proteobacteria(100)	Betaproteobacteria(100)	Burkholderiales(100)	Comamonadaceae(100)	Comamonas(100)
